# Glucocorticosteroid-sensitive inflammatory eosinophilic pseudotumor of the bladder in an adolescent: a case report

**DOI:** 10.1186/1752-1947-3-136

**Published:** 2009-11-19

**Authors:** Danfeng Xu, Yushan Liu, Yi Gao, Xuezhi Zhao, Chuangyu Qu, Changlin Mei, Jizhong Ren

**Affiliations:** 1 Urology Department of Changzheng Hospital, Shanghai, 200003, PR China; 2 Kidney Diseases Institute of Changzheng Hospital, the Second Military Medical University, Shanghai, 200003, PR China

## Abstract

**Introduction:**

Inflammatory eosinophilic pseudotumor of the bladder is a rare inflammatory bladder disease. The etiology and pathophysiology of this condition are still unclear. Few case reports have described inflammatory eosinophilic pseudotumor of the bladder in adults or children. Although benign, this disease is occasionally clinically aggressive and locally invasive, thus open surgical removal or complete transurethral resection is recommended.

**Case presentation:**

We present the case of a biopsy-proven inflammatory eosinophilic pseudotumor of the bladder in a previously healthy 16-year-old male adolescent with 2-month history of frequent micturition and dysuria with no significant apparent causative factors. The tumor regressed after a 6-week course of glucocorticosteroids.

**Conclusion:**

To the best of our knowledge, our case is a rare case of inflammatory eosinophilic pseudotumor of the bladder treated with complete conservative management. Due to its glucocorticosteroid-sensitive nature, we postulate that this disease belongs to a subgroup of eosinophilic disorders.

## Introduction

Inflammatory eosinophilic pseudotumor (IEPT) of the bladder is a rare, benign and proliferative lesion of the submucosal stroma [1]. The first pediatric inflammatory eosinophilic bladder tumor was reported in 1960 and since then it has usually been termed eosinophilic cystitis [2]. A few case reports have described IEPT of the bladder in both adults and children. IEPT of the bladder presents as a generalized inflammation of the bladder as well as a localized bladder mass [3]. Despite the benign inflammatory process, malignant-appearing histologic features of IEPT of the bladder can require radical tumor resection [2]. We present a case of biopsy-proven IEPT of the bladder in an adolescent, with tumor regression after administration of glucocorticosteroids.

## Case presentation

**Figure 1:**
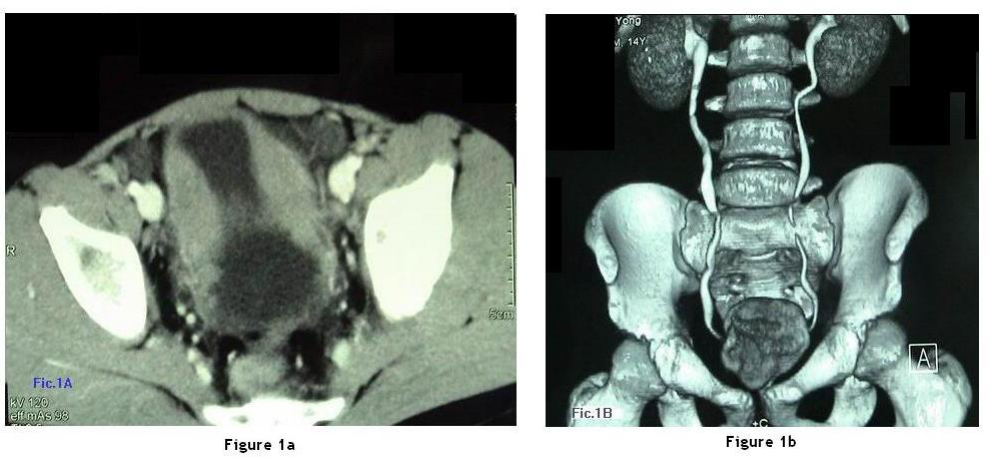
**A**. Computerized tomography of pelvis disclosed solid homogeneic-mass involving the wall of the dome and two lateral walls of the urinary bladder, average thickness of bladder wall was 1.9 cm. **B. **Computed tomography with three-dimensional imaging showed a normal upper urinary tract image with no nephrohydrosis, normal ureters and thickened walls of the bladder.

In September 2005, a previously healthy 16-year-old boy was admitted to a local hospital with 2-month history of frequent micturition and dysuria, but no apparent causative factors. The patient had no known history of drug allergies. Physical examination revealed a palpable suprapubic mass. Urine analysis showed no evidence of infection. An ultrasound examination revealed a bladder mass. Computed tomography (CT) of the abdomen and pelvis revealed a solid homogeneic mass involving the wall of the dome and two lateral walls of the urinary bladder; the average thickness of the bladder wall was 1.9 cm (Figure 1A). CT scan with three-dimensional imaging showed a normal upper urinary tract image with no nephrohydrosis, normal ureters and thickened walls of the bladder (Figure 1B).

Cystoscopy revealed homogeneic evagination of a solid submucosal mass at the dome, and two lateral walls of the bladder, but the mucosa on the tumor surface had integrity with no hemorrhagic spots. The patient was then transferred to our hospital. Results of a magnetic resonance imaging (MRI) scan were the same as the previous CT scan. Urodynamics indicated an unstable bladder with a volume of 70 ml. Reflection of the detrusor muscle was normal, as was coordination with the external sphincter, with no obstruction. Six tissue specimens were procured by transurethral resection on cystoscopy. The pathology report on the tissue biopsies indicated chronic inflammatory change with no evidence of malignancy, and a large quantity of eosinophilic cell suffusion infiltration accompanied by spindle cell proliferation. The blood count showed 1% eosinophil granulocytes, with an absolute value of 500/mm^3 ^(0.05×10^9^/l). The biochemistry tests did not show any abnormalities. Blood sedimentation rate was 11 mm/h and the level of serum C-reactive protein was 3.20 g/l. Serum levels of immunoglobulin (Ig)E, IgG, IgM and IgA1 were in the normal range. Occurrences of the lesion in other parts of the patient's body were excluded by physical examination and auxiliary examinations.

Prednisone, a glucocorticosteroid, was administered orally (20 mg/day), along with 200 mg (100 mg, twice a day for 1 week) of ranitidine to treat vomiting symptoms after the MRI scan. Two weeks later, the symptoms and frequency of dysuria had subsided and an ultrasound check showed that the average thickness of the bladder wall had decreased from 1.9 cm to 1.2 cm. The patient was discharged. Four weeks later, the average thickness of the bladder wall had further decreased from 1.2 cm to 0.8 cm. After 6 weeks of glucocorticosteroid therapy, the patient completely recovered, was asymptomatic and had a normal bladder. A follow-up 3 years later revealed an asymptomatic patient and ultrasonic inspection and cystoscopy showed no evidence of disease recurrence.

## Discussion

IEPT of the bladder is a benign proliferative lesion of the submucosal stroma, also regarded as a rare and poorly understood form of allergic cystitis, that is, eosinophilic cystitis [1]. Manifestations of eosinophilic cystitis are not specific and can mimic those of other inflammatory and malignant bladder disorders, and cannot be distinguished from malignant tumors of the bladder, either endoscopically or radiographically [3]. Pathology of tissue biopsy may be the main method for differential diagnosis [3,4].

Current treatment modalities include transurethral resection of the bladder lesion along with nonspecific medical therapy, such as nonsteroidal anti-inflammatory agents, steroids or antihistamines. Although benign, this disease is at times clinically aggressive and locally invasive [3,4]. The proliferative nature of the IEPT histopathology has led some doctors to recommend open surgical removal or complete transurethral resection for definitive treatment [5,6]. We report a case of IEPT of the bladder which regressed with prednisone administration alone.

The etiology of IEPT of the bladder is unclear [1-3]. Causative factors such as food allergens, parasites and drugs have been implicated in the genesis of this disease. Eosinophilic cystitis following Bacille Calmette-Guérin (BCG) therapy has also been described [6].

It is generally thought that IEPT belongs to the inflammatory pseudotumour (IPT) disease group [1]. Because our patient was sensitive to glucocorticosteroid therapy, it suggested to us that IEPT of the bladder should be classified as an eosinophilic disorder [7], generated according to the site of eosinophilic infiltration associated with organ damage and dysfunction. Eosinophilic disorders (or eosinophilia) are driven by allergen-activated T helper (TH)2 cells that generate large amounts of TH2 cytokines for example, interleukin (IL)-4, IL-5, IL-13 [8]. Eosinophilic disorders in children are associated with multiple diseases, most frequently with infections and allergies, in other anatomical locations including the lung, liver, eye orbit, heart, spleen and the genitourinary tract [7]. We therefore hypothesize that IEPT of the bladder might belong to a subgroup of eosinophilic disorders of this background. However, our patient had a normal serum IgE level with no evidence of any IgE-mediated mechanisms, and no relevant allergens were identified. There was also no direct evidence that any autoimmune mechanism contributed to the disease in this patient.

**Figure 2:**
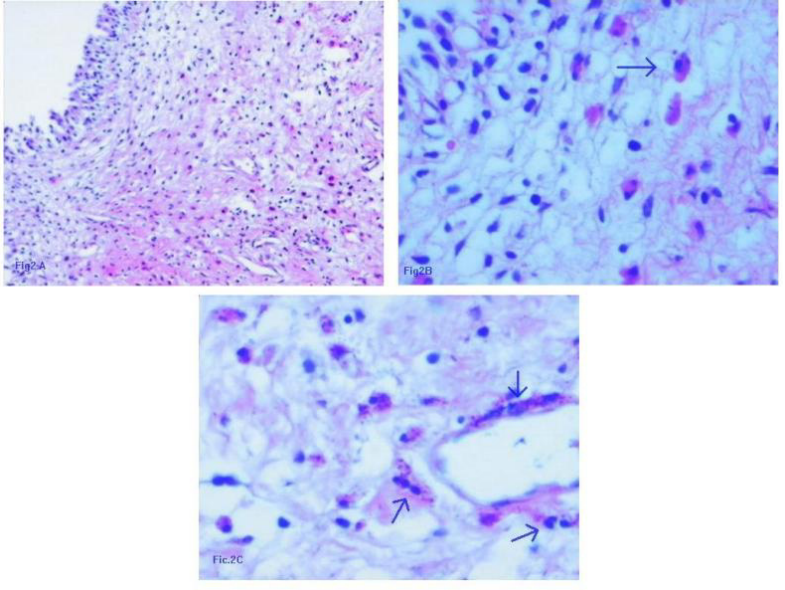
**Histopathology from a biopsy specimen demonstrates a great quantity of eosinophilic cell suffusion infiltration accompanied by proliferation of spindle cells (hematoxylin-eosin stain)**. **(A) **10× magnification fields under a light microscope. **(B) **40× magnification fields under a light microscope. An arrow shows a typical eosinophilic cell. **(C) **Arrows show infiltration of eosinophilic cells predominantly around the basement membrane of the microvessels in the proliferative tissue.

As the pathology test results show in Figure 2C, infiltration of eosinophilic cells was predominant around the basement membrane of the microvessels in the proliferative tissue. The patient had previous blood tests free from eosinophilia, so we postulate that local factors such as cytokines in the microenvironment of the bladder might have played an important role in the chemotactic immigration of the extrinsic source of the eosinophilic cells. This is in agreement with another study [7], which supposed that the clinical value of eosinophil counts is limited because of the unknown function of the eosinophils in the pathogenesis of most eosinophilic diseases.

## Conclusion

To the best of our knowledge, this case of an inflammatory pseudotumor of the bladder is one of only a few cases reported in the literature that have been treated with complete conservative management [9]. According to the glucocorticosteroid-sensitive nature of the condition, we postulate that this disease might belong to a subgroup of eosinophilic disorders.

## Abbreviations

BCG: Bacille Calmette-Guerin; CT: computed tomography; IEPT: inflammatory eosinophilic pseudotumor; Ig: immunoglobulin; IL: interleukin; IPT: inflammatory pseudotumour; MRI: magnetic resonance imaging; TH: T helper;

## Consent

Written informed consent was obtained from the patient for publication of this case report and any accompanying images. A copy of the written consent is available for review by the Editor-in-Chief of this journal.

## Competing interests

The authors declare that they have no competing interests.

## Authors' contributions

YL and DX managed the patient and reviewed the literature. YG and JR were the main writers of the manuscript. CM, XZ and CQ moderated the manuscript. All authors read and approved the final manuscript.
